# Research on Raman Spectral Denoising and Baseline Correction Method Based on Dynamic Mask Partition U-Net

**DOI:** 10.3390/molecules31214030

**Published:** 2026-11-09

**Authors:** Yihao Lin, Xiaoyue Xia, Yifan Zhang, Zheer Huang, Xuqing Wang, Yingsheng He, Jiye Wang, Yazhou Qin

**Affiliations:** 1 Key Laboratory of Drug Prevention and Control Technology of Zhejiang Province, Zhejiang Police College, 555 Binwen Road, Binjiang District, Hangzhou 310053, China; 2 School of Pharmacy, Hangzhou Normal University, Hangzhou 311121, China; 3 Key Laboratory of Drug Prevention and Control Technology of Zhejiang Province, National Narcotics Laboratory Zhejiang Regional Center, 555 Binwen Road, Binjiang District, Hangzhou 310053, China

**Keywords:** Raman spectroscopy, denoising, convolutional neural network, baseline correction

## Abstract

Raman spectroscopy, as a core technique for qualitative analysis of material structure and quantitative analysis of composition, is widely applied in fields such as materials science, biomedicine, and environmental monitoring. However, Raman spectral signals generally suffer from weak intensity and low signal-to-noise ratio, and are susceptible to interference from fluorescence background and instrument electronic noise, leading to baseline drift, blurred or even disappearing characteristic peaks. Traditional denoising methods mostly adopt a single smoothing strategy or global processing mode, making it difficult to adapt to the differentiated denoising requirements of different spectral regions, and often resulting in problems such as peak distortion or insufficient noise suppression. To this end, this study proposes a joint preprocessing method based on dual-Mask peak-Aware U-Net joint denoising and baseline correction (DMPA-UNet). First, a lightweight convolutional network is used to generate an adaptive mask, dynamically dividing the spectrum into peak regions and non-peak regions; then, a dual-branch U-Net is adopted to process the two regions differently, and mask-weighted fusion is used to obtain the denoised spectrum; finally, an independent U-Net is used to perform global baseline estimation and removal, achieving collaborative optimization of denoising and baseline correction. Comparative experiments between this method and other methods, such as SG (Savitzky–Golay) filtering, median filtering, and CNNs (Convolutional Neural Networks), show that, compared with the original noisy spectrum, this method reduces MSE by approximately 80.9%, improves SNR (signal-to-noise ratio) by approximately 116.0%, and improves PSNR (peak signal-to-noise ratio) by approximately 38.9%; the KNN (K-Nearest Neighbor) classification accuracy can reach 84.5%, an improvement of 69.5% over the original noisy spectrum. This method balances peak preservation and noise suppression, providing an effective preprocessing scheme for qualitative analysis of Raman spectra.

## 1. Introduction

Raman spectroscopy, as a non-destructive analytical technique based on the inelastic scattering of molecular vibrational and rotational energy levels, can provide unique molecular fingerprint information and is one of the core methods for material structure identification and compositional quantitative analysis [1]. With its unique advantages, such as high specificity, label-free detection, and insensitivity to water, Raman spectroscopy has demonstrated irreplaceable application value in numerous fields, including materials science, biomedicine [2], and environmental monitoring [3,4,5,6,7]. However, the Raman scattering effect is extremely weak, resulting in low intensity and poor SNR of the acquired raw spectral signals [8,9]. The signal generated during the measurement process is highly susceptible to severe interference from multiple factors: the fluorescence background from the sample itself introduces a slowly varying broadband baseline drift [10]; the detector’s readout noise, dark current noise, and ambient stray light, among others, superimpose as high-frequency random noise [11]; and cosmic rays striking the detector also produce random sharp pulse peaks. These interference factors are coupled with one another, so that the raw Raman spectra are often accompanied by baseline distortion and buried or even deformed characteristic peaks, severely affecting the accuracy of subsequent qualitative identification [12,13].

To address the above issues, researchers have developed various Raman spectral denoising methods over the years. Traditional methods are represented by SG filtering, which suppresses noise through local polynomial fitting within a sliding window [14]. Gil et al. trained a single-layer convolutional model and compared it with SG filtering, finding that the neural network performed better in the SNR range of 2–5.5 [15]. However, such methods essentially adopt a globally uniform processing strategy, making it difficult to adapt to the differentiated requirements of different spectral regions, and parameter selection is highly dependent on experience.

Among transform-domain-based methods, wavelet transform is a typical denoising method. The iterative differential autoregressive spectral estimation method proposed by Guo et al. achieved SNR improvement on samples such as Teflon and azithromycin [16]. Bian et al. applied variational mode decomposition to Raman spectroscopy and achieved better results than SG filtering on simulated data [17]. However, the performance of such methods is highly dependent on parameters such as the choice of wavelet basis, the number of decomposition levels, and threshold settings, and artifacts are easily introduced when dealing with non-stationary signals.

With the development of deep-learning technology, spectral denoising methods based on CNN have attracted considerable attention due to their powerful nonlinear feature-extraction capabilities. Barton et al. trained a CNN with simulated spectra and significantly improved the quality of spectra with low SNRs [18]. Zeng et al. introduced multi-scale feature fusion blocks into U-Net, reducing the integration time from 3 s to 1 s in Raman imaging of cervical cancer cells while still obtaining clear images [19]. The self-supervised U-Net denoising method proposed by Wu et al. increased the SNR by more than a factor 4 in experiments [20]. Kazemzadeh et al. validated the superiority of cascaded deep convolutional neural networks (CNNs) based on ResNet or U-Net architectures on bladder cancer-tissue spectra [21]. The masked autoencoder self-supervised learning method proposed by Ren et al. achieved over 80% clustering accuracy without the need for labeled data [22]. Parallel to denoising, deep learning has also been applied to baseline correction: Chen et al. proposed a triangular deep convolutional network (TDCN) that integrates the design concepts of ResNet and U-Net, achieving higher baseline correction accuracy, shorter computation time, and better preservation of peak intensity and shape than existing methods [23]. Atia et al. developed a dual-branch U-Net in which a shared encoder diverges into two decoding heads, one for the Raman signal and one for the baseline, coupled through a cross-attention gating mechanism, thus enabling simultaneous denoising and baseline correction of microplate Raman spectra [24]. Zhao et al. trained a one-dimensional transformer on manually annotated data and achieved lower MAE and SAM scores than CNN and ResUNet [25], and Innocente et al. proposed the IS (Integrity Spectrum) Score, a quantitative and automatic metric that evaluates baseline correction quality without requiring ground truth labels or manual inspection [26]. However, research by Mokari et al. demonstrated that the diversity of training data significantly affects the model’s robustness to noise [27].

However, existing denoising methods generally face the trade-off dilemma between peak preservation and noise suppression. Although traditional methods such as SG filtering can suppress noise, their parameter selection is highly dependent on experience and can easily lead to distortion of weak peaks [1]; the performance of methods such as wavelet transform depends on the selection of parameters such as basic functions and thresholds. Deep-learning methods that have emerged in recent years have shown performance superior to traditional methods, but their convolution kernels slide and compute in the same way across all spectral regions, making it difficult to adaptively adjust processing strategies according to the characteristics of different regions. To relieve this trade-off, Lee and co-worker assigned a different window value to each region according to the detected peaks, which removed noise while preserving the height and shape of the peaks [28]. The division of regions, however, still relies on a peak detection step to determine where each window applies.

To address the above problem, this study proposes a Raman spectral preprocessing method based on dual-mask peak-aware U-Net joint denoising and baseline correction (DMPA-UNet). The core innovations of this method are as follows. Firstly, a lightweight convolutional network is used to generate adaptive masks, dynamically dividing the spectrum into peak regions and non-peak regions. Second, a dual-branch U-Net structure is designed, in which a weak denoising strategy is adopted for peak regions to preserve peak shape, while strong denoising is applied to non-peak regions to effectively suppress noise, and mask-weighted fusion is used to achieve region-specific fine processing. Finally, an independent U-Net is introduced for global baseline estimation and subtraction, enabling collaborative optimization of denoising and baseline correction. The experimental results show that, compared with the original noisy spectrum, the MSE of this method is reduced by approximately 80.9%, down to 0.0027; the SNR is improved by approximately 116.0%, reaching 13.6 dB; and the PSNR is improved by approximately 38.9%, reaching 26.1 dB. In the KNN classification task, the classification accuracy can reach 84.5%, an improvement of 69.48% over the original noisy spectrum. These results verify the significant advantages of this method in balancing peak protection and noise suppression and in improving the accuracy of qualitative analysis of Raman spectra.

## 2. Results

### 2.1. Spectral Analysis

**Figure 1:**
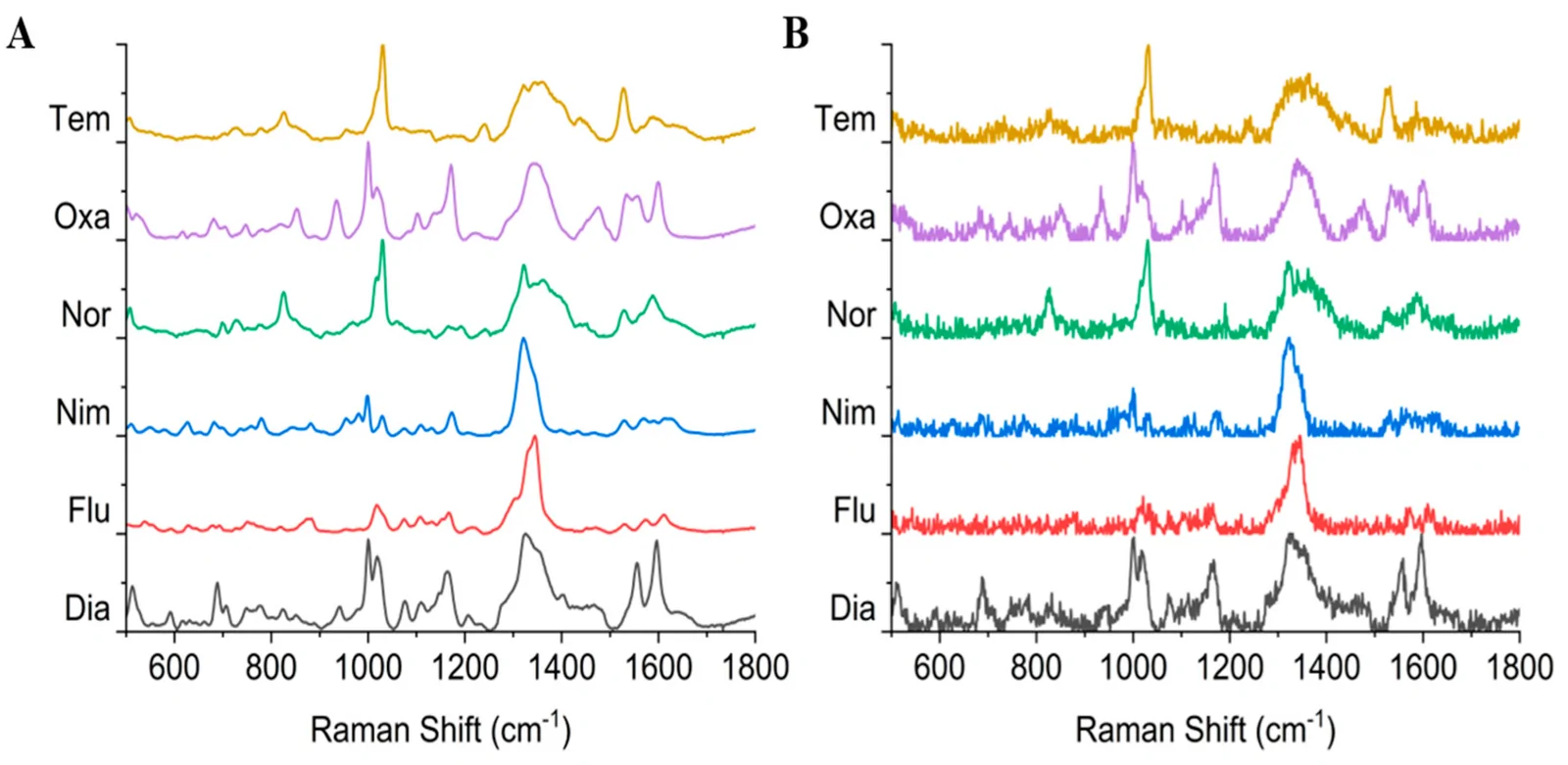
Original (**A**) and with artificially added noise (**B**) Raman spectra of six benzodiazepines.

As shown in Figure 1A, the six benzodiazepines exhibit distinct Raman spectral fingerprints. The characteristic Raman peak positions for each substance are summarized in Table 1. The test results of 6 substance are shown in the Supplementary Materials. In Figure S1, A1–F1 show the molecular structures of six benzodiazepine drugs, A2–F2 present the characteristic peaks of the Raman, SERS, and DFT-calculated Raman spectra of the six substances, A3–F3 are the SERS spectra measured at different concentrations for each substance, and A4–F4 are the corresponding waterfall plots. The assignment of characteristic peak vibration modes for each substance is shown in Tables S1–S6.

**Figure 2:**
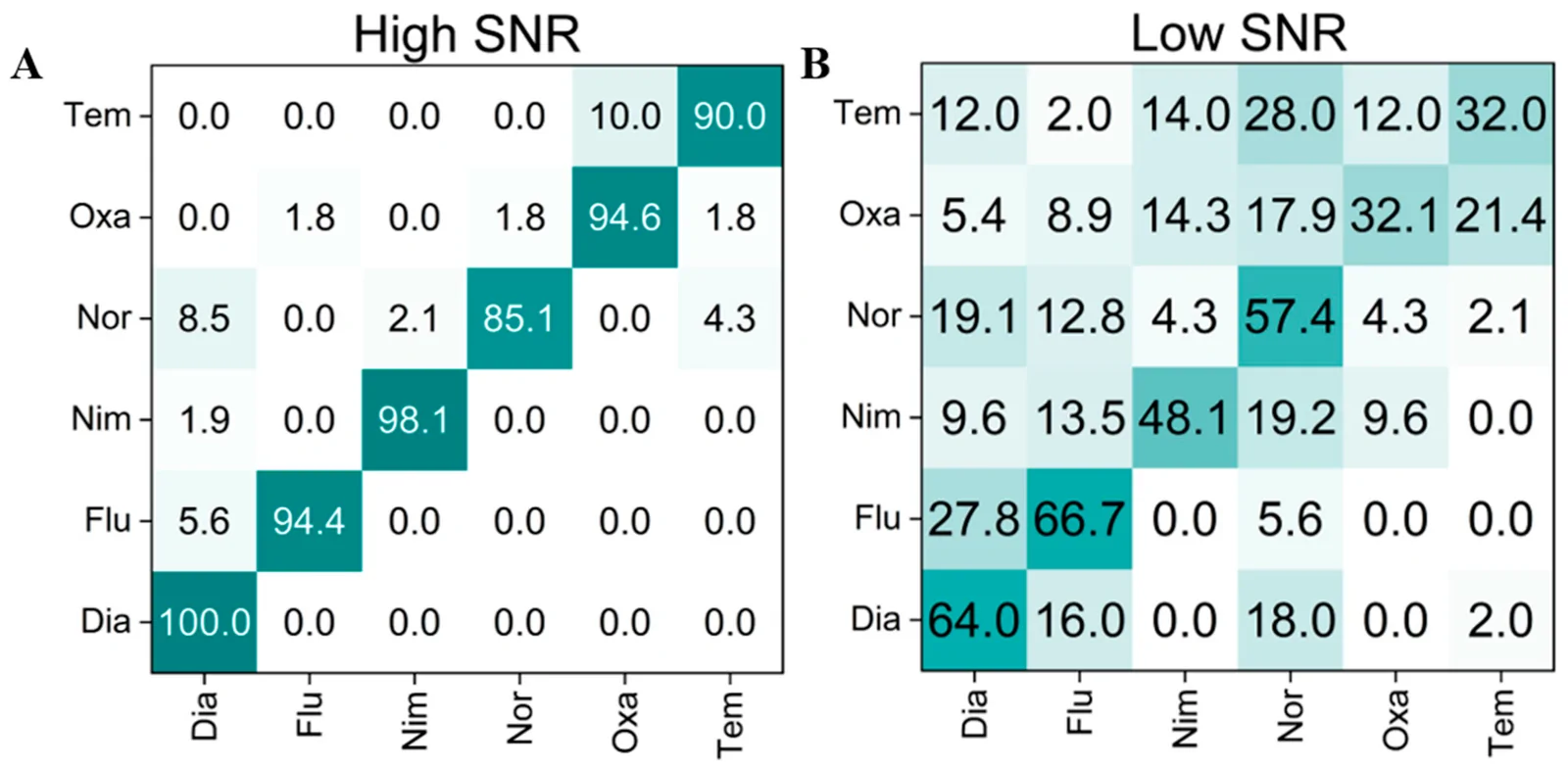
Confusion matrix of classification model on high-SNR (**A**) and low-SNR (**B**) spectral data.

We use KNN classifier to classify the spectra with high SNR and low SNR respectively. As can be seen from Table 2, the artificially added noise disrupted the spectral information, resulting in a decrease in classification accuracy. The confusion matrix in Figure 2 illustrates that noise causes severe inter-class confusion, which also explains the reason for the low accuracy under low SNR in Table 2.

### 2.2. Dual-Mask Peak-Aware U-Net Joint Denoising and Baseline Correction Method

**Figure 3:**
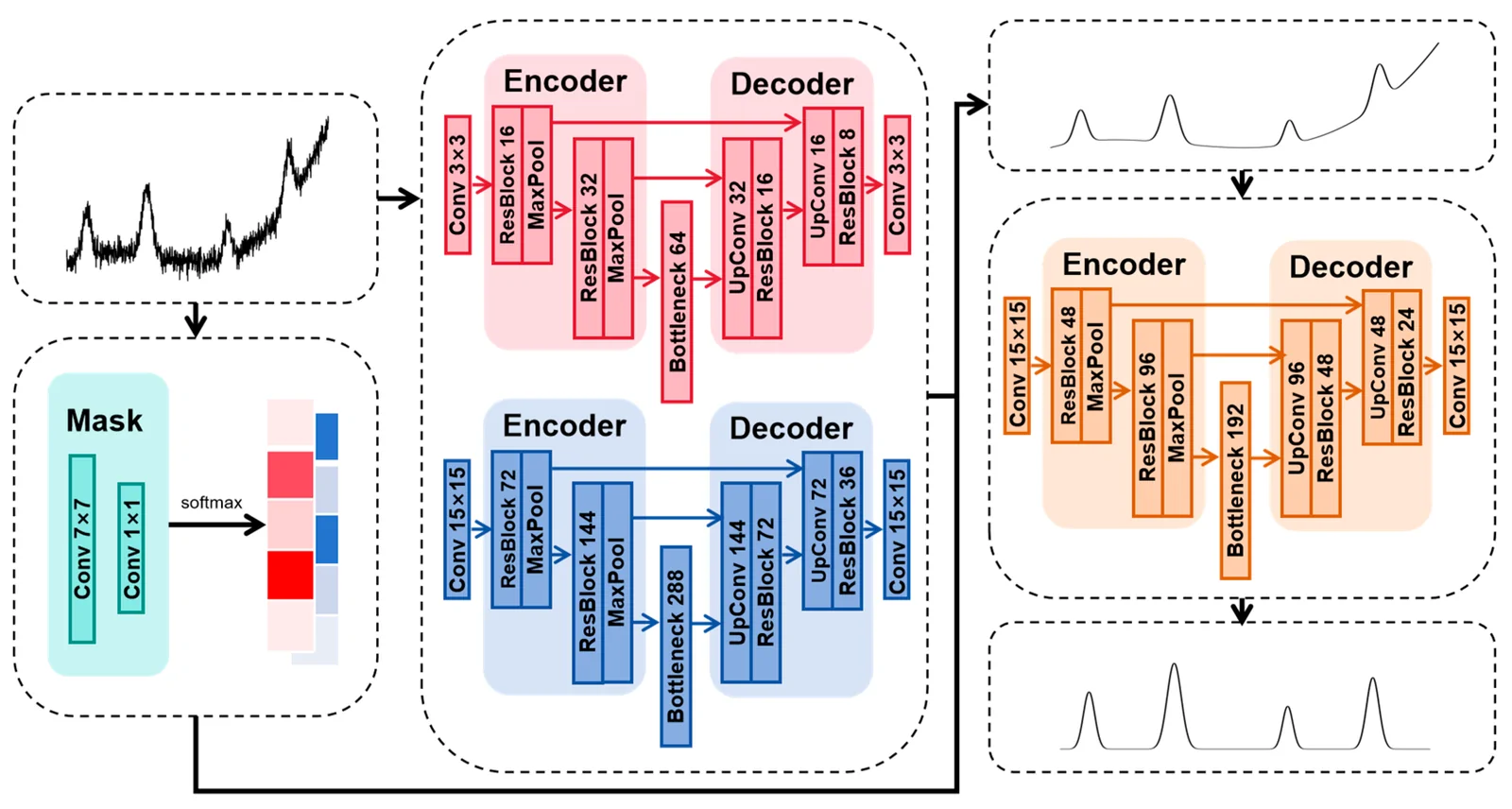
Overall framework of DMPA-UNet.

The overall framework of the proposed DMPA-UNet in this study is shown in Figure 3, which mainly consists of three core modules: a mask generation network, a dual-branch denoising network, and a baseline correction network. Its basic processing flow is as follows: taking spectrum as input, a mask generation network first outputs probability masks for peak and non-peak regions; then the spectrum and masks are jointly fed into a dual-branch U-Net. Finally, the U-Net is used to estimate and subtract the residual baseline drift, and output the spectrum.

#### 2.2.1. Mask Generation

**Figure 4:**
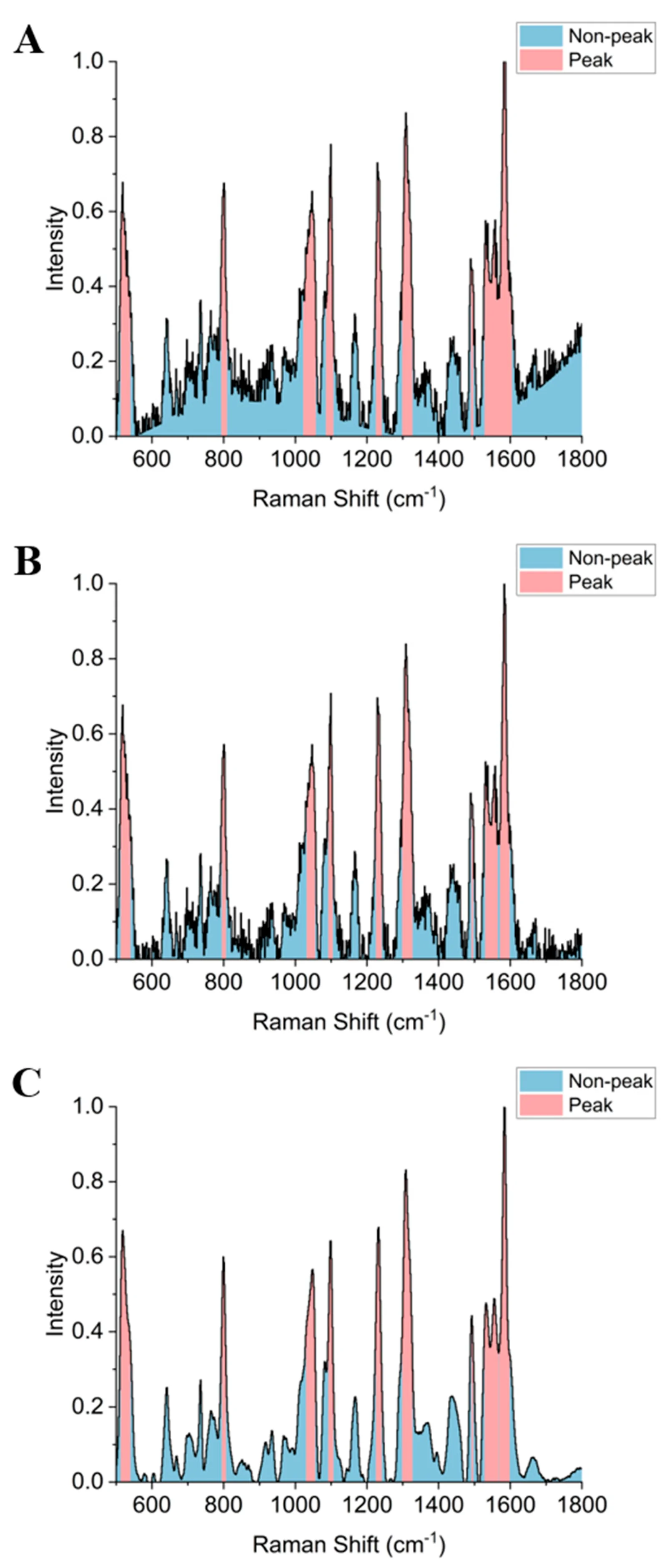
Spectral mask division in different preprocessing stages: (**A**) Original spectrum, (**B**) after baseline correction, and (**C**) after baseline correction and smoothing.

As shown in Figure 4, the mask generation network takes spectrum as input and aims to achieve adaptive partitioning of the spectral regions. The network first extracts the global profile and low-frequency trend of the spectrum through a wide convolutional kernel layer, while expanding the single-channel input to 16 channels to enhance feature representation capability. Subsequently, after batch normalization and ReLU activation, the channels are compressed back to a single channel through a 1 × 1 convolution, and a Sigmoid activation function is used to output the mask, producing the probability that each wavenumber point belongs to a peak region.

#### 2.2.2. Denoising

**Figure 5:**
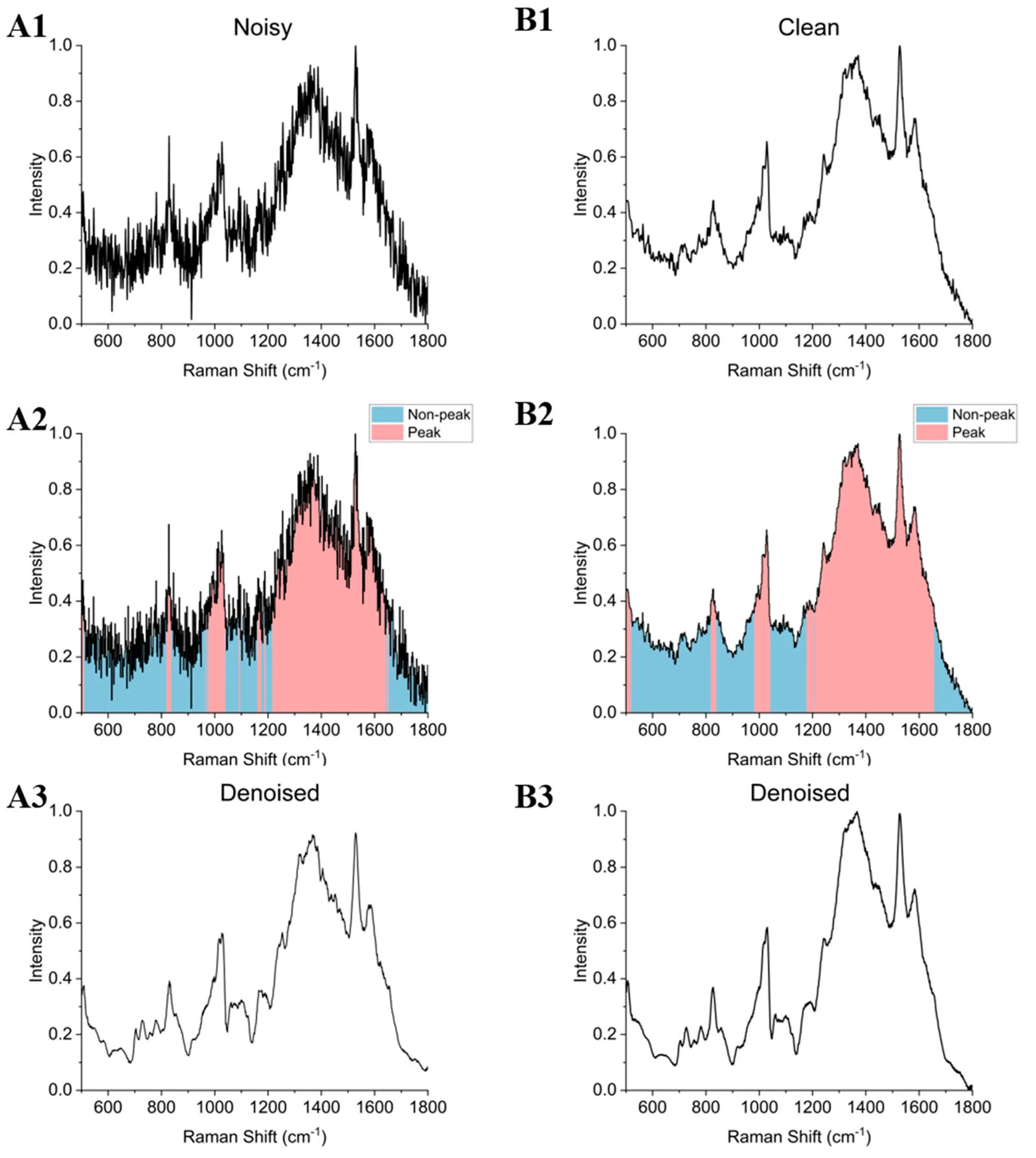
Denoising process and results of the dual-branch U-Net: (**A1**) Noisy spectrum; (**A2**) noisy spectrum mask division; (**A3**) denoised spectrum; (**B1**) clean spectrum; (**B2**) clean spectrum mask division; and (**B3**) denoised spectrum.

The denoising network adopts a dual-branch U-Net architecture, applying differentiated processing to peak regions and non-peak regions respectively. Both branches are based on an encoder–decoder structure and pass the spatial details of each encoder layer to the decoder through skip connections to preserve the local features of the spectrum. Among them, the peak region branch uses smaller convolution kernels to protect the peak height, width, and shape of characteristic peaks; the non-peak region branch uses larger convolution kernels and implements a strong denoising strategy to effectively suppress random noise in non-peak regions. After the two branches respectively output the regionalized preliminary denoising results, they are element-wise multiplied with the mask output by the mask generation network and weighted-summed to obtain the fused denoised spectrum. Figure 5 shows the denoising process and results of the dual-branch U-Net; it can be seen that the random noise in the noisy spectrum is significantly suppressed, while the characteristic peaks are well preserved.

#### 2.2.3. Baseline Correction

**Figure 6:**
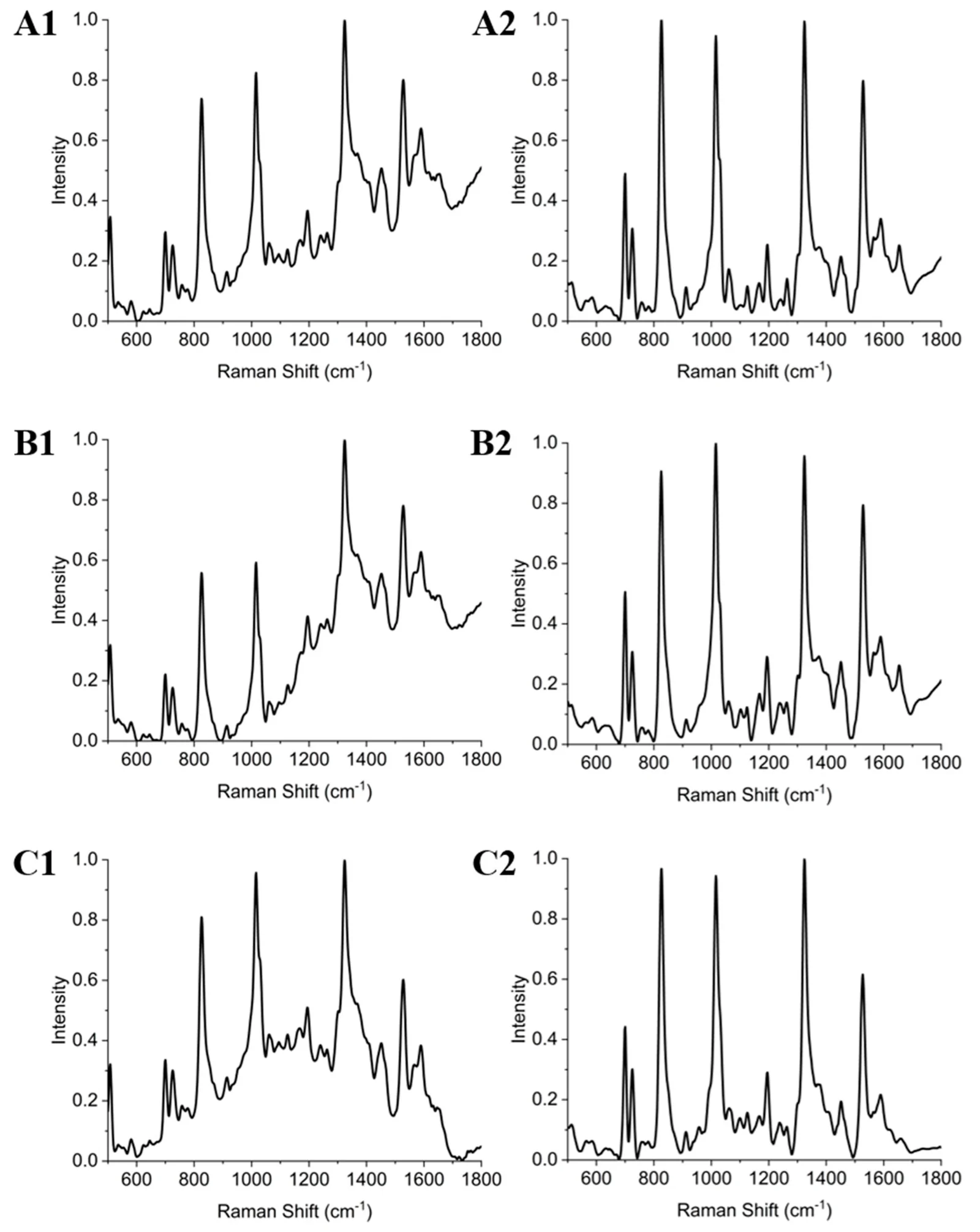
Correction effects under different artificial baseline drift patterns: (**A1**) Linear slope, (**B1**) triangular shape, and (**C1**) sigmoid curve; and their corresponding corrected spectra (**A2**–**C2**).

To evaluate the robustness of the model against varying baseline drifts, four typical artificial baseline patterns were superimposed on the clean spectra: a linear slope, a triangular shape, a sigmoid curve, and a condition without baseline. To highlight the influence of the baseline, the clean spectral signal was compressed to 0.60 of its original amplitude before adding the baselines. The maximum amplitude of the artificial baselines was uniformly set to 0.35. Figure 6 shows the correction results under these different baseline drift patterns. It can be seen that the residual baseline drift is effectively removed and the characteristic peaks are well restored, without obvious distortion.

The spectra denoised by the DMPA-UNet were compared with those obtained by traditional denoising methods and deep-learning denoising methods, and the results are shown in Table 3.

**Figure 7:**
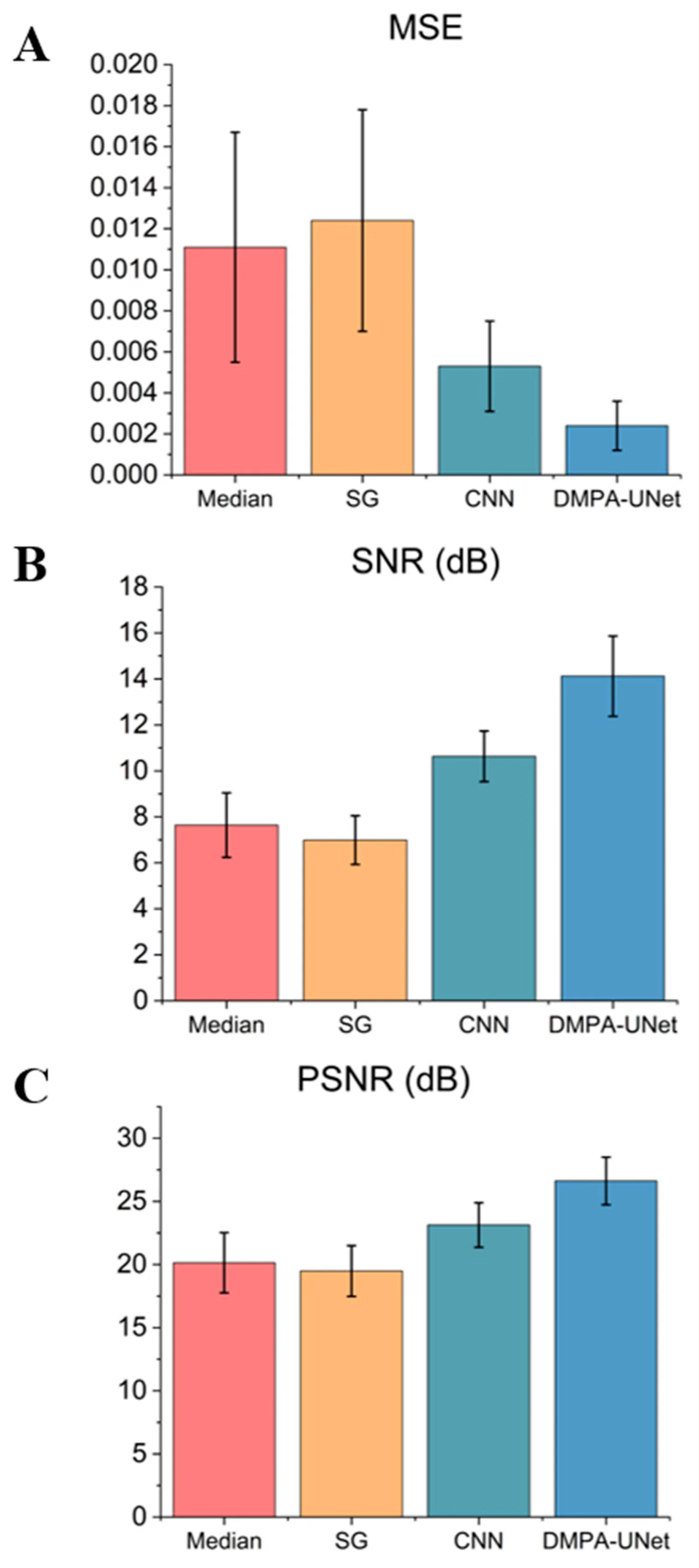
Comparative visualization of different methods on MSE (**A**), SNR (**B**), and PSNR (**C**) indicators.

As shown in Table 3, DMPA-UNet outperforms the other comparison algorithms in all three metrics: MSE, SNR, and PSNR. Figure 7 provides an intuitive comparison of three metrics, showing that DMPA-UNet achieves the lowest MSE and the highest SNR and PSNR among all methods. Although median filtering, SG filtering, and CNN can suppress the noise in the original noisy spectra to a certain extent, they still have limitations in terms of denoising amplitude and characteristic peak preservation. The DMPA-UNet model achieved the best overall performance: MSE was as low as 0.0027, reduced by approximately 80.9% compared with the original noisy spectrum, and reduced by approximately 75.5%, 73.5%, and 49.1% compared with median filtering, SG filtering, and CNN, respectively, with the overall spectral morphology well restored; SNR reached 13.6 dB, improved by approximately 116.0% compared with the original noisy spectrum, and improved by approximately 77.5%, 68.5%, and 27.7% compared with median filtering, SG filtering, and CNN, respectively, indicating that noise was significantly suppressed; PSNR reached 26.1 dB, improved by approximately 38.9% compared with the original noisy spectrum, and improved by approximately 29.5%, 27.0%, and 12.8% compared with median filtering, SG filtering, and CNN, respectively, indicating that the model retained the peak height and peak shape of characteristic peaks well while denoising. After processing with DMPA-UNet, random noise was effectively suppressed, and the key characteristic peak positions and profiles of the six benzodiazepines remained intact.

### 2.3. Classification Performance

To verify whether the DMPA-UNet denoising algorithm weakens the characteristic peaks of Raman spectra during denoising, this study used the spectra denoised by each method as feature inputs and employed a KNN classifier for substance classification. The results are shown in Table 4.

**Figure 8:**
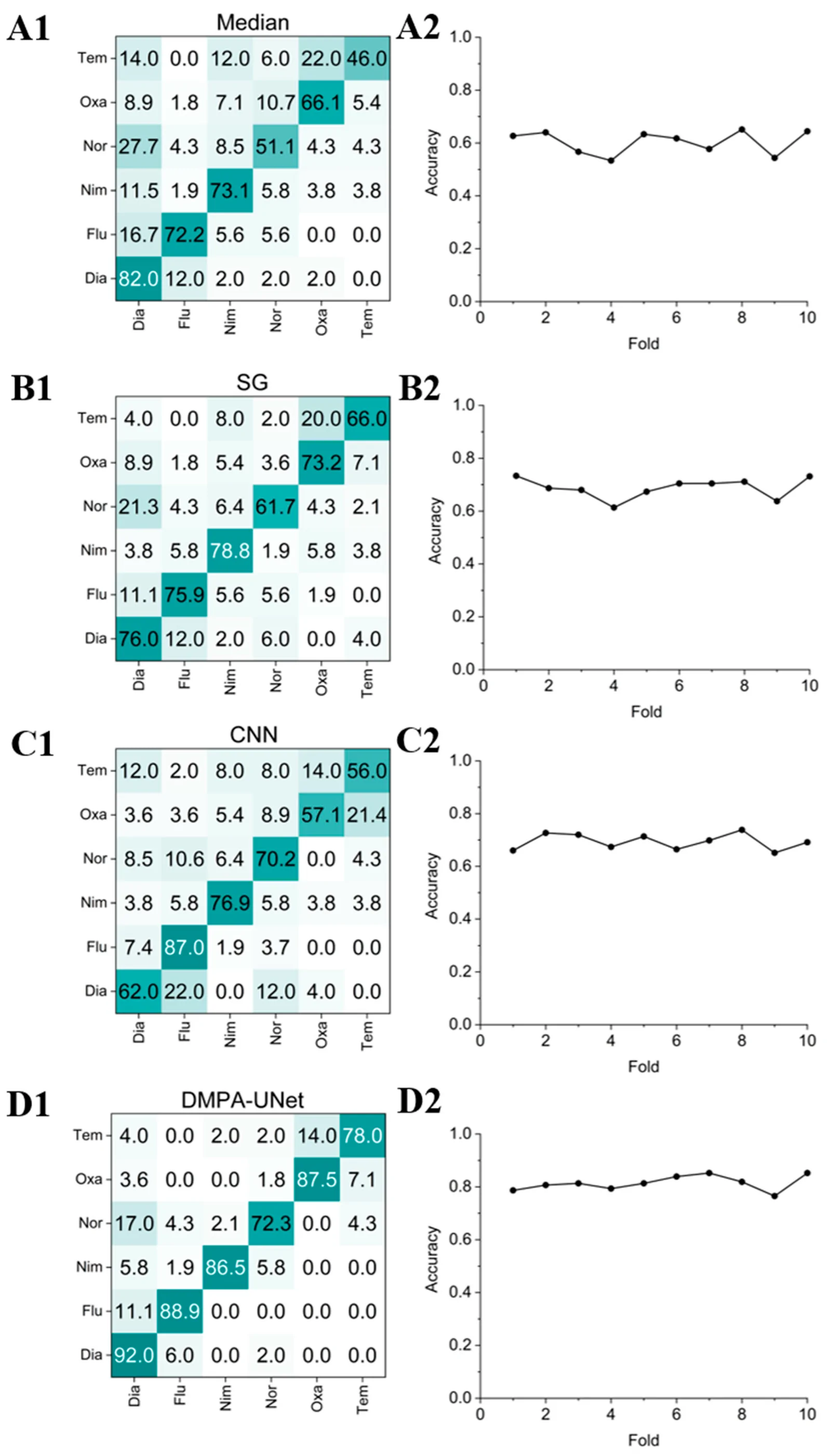
KNN classification performance under different noise reduction methods.

**Figure 9:**
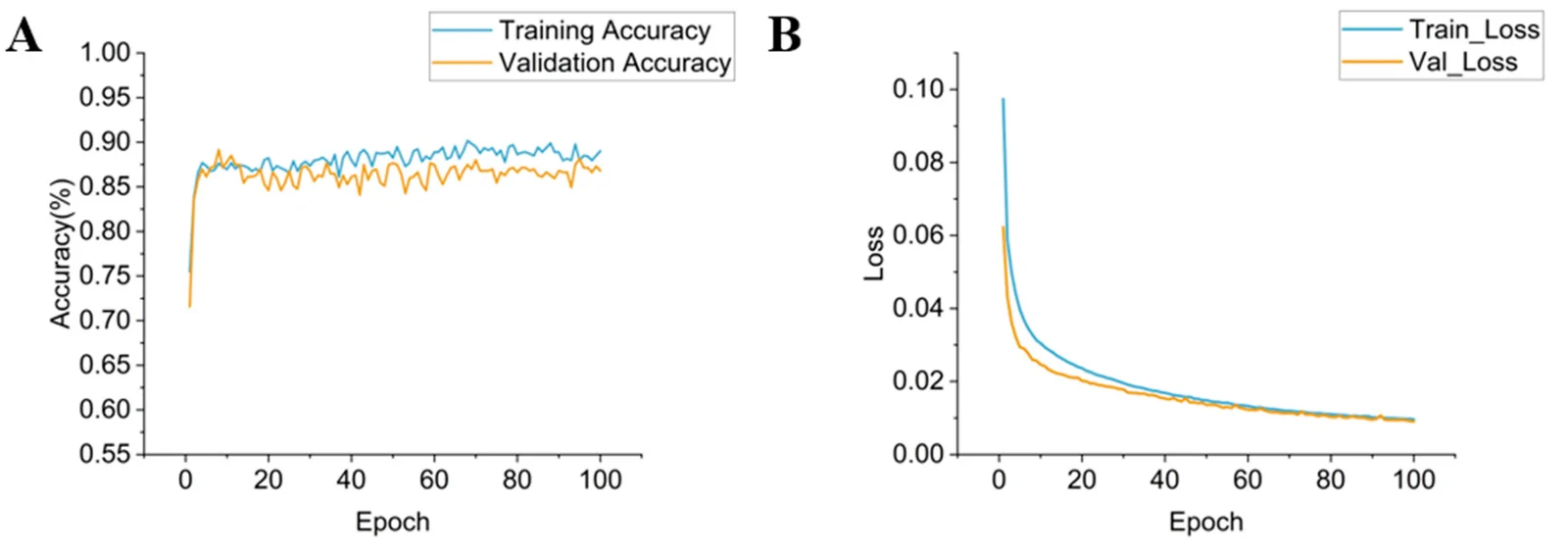
Model training and validation curve: Accuracy (**A**) and loss (**B**).

As shown in Table 4, the raw noisy spectra exhibit the worst classification performance across all metrics, serving as the baseline. Although conventional filtering methods such as median filtering and SG filtering improve classification results to some extent by suppressing random noise, they struggle to fully restore discriminative spectral features. Among these, SG filtering generally outperforms median filtering due to its superior local polynomial fitting. The conventional CNN method shows a moderate improvement over median filtering, yet its performance still lags behind SG filtering, suggesting that critical spectral discriminative information may be lost during the global feature-extraction process. In contrast, the proposed DMPA-UNet achieves the most significant improvement, with a classification accuracy reaching 84.47%, substantially outperforming all other techniques. This demonstrates that the dynamic mask partitioning strategy effectively balances strong denoising with peak shape preservation, providing highly robust feature inputs for downstream tasks. According to Figure 8, the confusion matrix and ten-fold cross-validation results confirm this superiority, indicating that, compared with conventional methods, DMPA-UNet substantially reduces sample misclassification and maintains highly stable accuracy across different folds. As shown in Figure 9, the training and validation curves converge stably without obvious overfitting, further confirming the reliability of the model training.

## 3. Discussion

This study proposes a DMPA-UNet preprocessing method for joint denoising and baseline correction of Raman spectra. This method integrates dynamic mask partitioning with a dual-branch U-Net architecture, aiming to address the inherent contradiction between noise suppression and feature peak fidelity in traditional global preprocessing algorithms. A dataset was constructed based on SERS spectra of six individual benzodiazepine substances to validate the effectiveness of the proposed method.

Comparative experiments demonstrate that DMPA-UNet achieves the best performance across all denoising evaluation metrics, significantly outperforming median filtering, SG filtering, and conventional CNN. Its core advantage lies in the dynamic masking mechanism, which effectively avoids the peak shape distortion caused by traditional smoothing algorithms, allowing the denoised spectra to achieve a better balance between fidelity and signal-to-noise ratio. In downstream qualitative classification tasks, the classification accuracy of spectra preprocessed by DMPA-UNet has been significantly improved. Confusion matrices and cross-validation results further confirm that this method can effectively reduce the misclassification rate of samples, providing highly stable feature inputs for qualitative identification.

Although this method performs excellently in qualitative identification of single substances, we must still objectively examine its advantages, limitations, and generalizability. The advantage of this method lies in achieving both strong denoising and peak shape preservation through differentiated processing. However, this dual-mask mechanism is highly sensitive to local high-frequency variations. For broad shape features lacking clear boundaries, due to their low-frequency characteristics that highly overlap with the slowly varying fluorescence baseline, the mask network may struggle to accurately distinguish signal from background. In such cases, the algorithm carries the risk of misclassifying broad peaks as baseline, leading to excessive subtraction. Furthermore, since the present study has only been evaluated on single-component solutions, the generalization capability of this method for multicomponent complex mixtures with severe spectral overlap still requires further validation.

In summary, DMPA-UNet provides an advanced solution for Raman spectral preprocessing of spectra with prominent characteristic peaks, but it should be applied with caution when dealing with broadband spectra or complex mixtures. Future work will focus on incorporating frequency-domain feature information to better distinguish broadband signals from baselines, and extending the dataset to multi-component mixtures to further enhance the model’s generality and robustness.

## 4. Materials and Methods

### 4.1. Materials and Equipment

The SERS detection materials were oxazepam, diazepam, flunitrazepam, nimetazepam, nordazepam, temazepam, six standard powders of benzodiazepines. We used 2000 ppm solutions of these six substances, adding ultrapure water to prepare a total of 30 solutions at 5 concentrations: 0.01 ppm, 0.001 ppm, 0.1 ppb, 0.01 ppb, and 0.001 ppb; NaCl solution; Au NCs.

SERS detection instrument: Portable Raman detector PERS-F700 was purchased from Pushi Nano Technology Co., Ltd. in Xiamen, China. Excitation wavelength of 785 nm, laser power at 100%, and integration time of 1000 ms. The spectral range is −1019–3349 cm^−1^.

### 4.2. Raman Spectroscopy Detection

Step 1: Prepare the detection solution. Use a 20–200 μL pipette to take 50 μL of the sample solution to be tested (specifically: five solutions with concentration gradients of 0.01 ppm, 0.001 ppm, 0.1 ppb, 0.01 ppb, and 0.001 ppb), 50 μL of NaCl solution, and 100 μL of Au NCs solution into a SERS tube, and mix by shaking with hand.

Step 2: Detection. Place the SERS tube containing the detection solution into the portable Raman detector PERS-F700, use the detection center-self-built library module to establish a new library, adjust the detection parameters, set the integration time to 1000 ms, adjust the laser power to 100%, and save after spectral acquisition. Afterwards, perform duplicate detection operations in the established library. For each type of detection sample, multiple sets of data are collected to observe data deviation, and experimental data with good repeatability are selected. After detection is completed, the data are exported and saved.

### 4.3. Data Preprocessing

The Raman spectral fingerprint region of 500–1800 cm^−1^ was intercepted, the number of sampling points was unified to 820, linear interpolation was used to align the standard wavenumber axis, and the spectral intensity was normalized to the [0, 1] interval. Five concentration gradients were prepared by performing a 10-fold serial dilution from the 2000 ppm stock solution using ultrapure water. A total of 1646 valid Raman spectra were collected, and the number of recorded spectra for each substance at different concentrations is shown in Table 5. A total of 1646 valid Raman spectra were collected, and the distribution of samples for each substance is shown in the table below.

### 4.4. Network Structure

The kernel size for median filtering was set to 11. The window length for SG filtering was set to 21, and the polynomial order was set to 1.

To ensure a fair comparison, all comparison methods used exactly the same ALS baseline correction parameters: smoothing parameter, λ = 10^5^; asymmetry parameter, *p* = 0.01; and the number of iterations was 10.

The CNN model consists of an input convolutional layer (1 to 32 channels), 4 residual blocks (each containing two 3 × 1 convolutional layers and an identity skip connection), a channel attention module (with a reduction ratio of 4), and a global residual output with a learnable scaling factor.

The proposed DMPA-UNet was implemented in PyTorch (version 2.9.1), running on the Windows operating system. All experiments were conducted on a general-purpose laptop equipped with an Intel(R) Core (TM) Ultra 5 125H CPU and 32 GB RAM. During training, the batch size is set to 32, and the total number of training epochs is 100. The Adam optimizer is adopted, with an initial learning rate of 1 × 10^−4^. During training, the ReduceLROnPlateau scheduler (decay factor = 0.5; patience = 5) is used to dynamically adjust the learning rate. The loss function weights are set to λ_1_ = 0.3, λ_2_ = 0.1, and λ_3_ = 0.02, respectively. The dropout rate is set to 0.05. The dataset is split into training, validation, and test sets in a 7:2:1 ratio. On the training set, we performed data augmentation by adding Gaussian random noise (standard deviation of 0.08) and simulated baseline drift (amplitude of 0.08); the validation and test sets remained unaugmented to ensure objective evaluation. In the downstream KNN classification task, the number of neighbors was set to k = 5, and ten-fold stratified cross-validation was used for performance evaluation.

#### 4.4.1. Loss Function

The loss function is composed of a combination of the global reconstruction loss and the weighted reconstruction loss for each region. The formula is as follows:

(1) $$L_{\mathrm{total}}=\lambda_{1}\cdot L_{L_{1}}+\lambda_{2}\cdot L_{\mathrm{region}}+\lambda_{3}\cdot L_{\mathrm{entropy}},$$

where $L_{L_{1}}$ is the global $L_{L_{1}}$ loss, used to supervise the global reconstruction accuracy of Raman spectra. The formula is as follows:

(2) $$L_{L_{1}}=\frac{1}{B\cdot L}\sum_{b=1}^{B}\sum_{i=1}^{L}\left|{\hat{y}}_{b,i}-y_{b,i}\right|,$$

where *B* is the batch size, and *L* is the number of spectral sampling points.

ℒ*_region_* is the region weight loss, used to constrain the denoising strength for different regions. The formula is as follows:

ℒ*_region_* = *α* ⋅ MSE*_peak_* + *β* ⋅ MSE*_bg_*, (3)

The MSE calculation formula for each region is as follows:

(4) $${\mathrm{MSE}}_{\mathrm{region}}=\frac{1}{B\cdot L}\sum_{b=1}^{B}\sum_{i=1}^{L}\left[m_{\mathrm{region},b,i}\cdot {\left({\hat{y}}_{b,i}-y_{b,i}\right)}^{2}\right],$$

ℒ_entropy_ is the mask entropy regularization loss, used to constrain the mask distribution. The formula is as follows:

(5) $$L_{\mathrm{entropy}}=-\frac{1}{B\cdot L}\underset{b,i}{\sum }[M_{\mathrm{peak}}\log(M_{\mathrm{peak}}+\varepsilon )+(1-M_{\mathrm{peak}})\log(1-M_{\mathrm{peak}}+\varepsilon )],$$

where *m_region_*_,*b*,*i*_ is the corresponding region mask, and *M_peak_* represents the probability mask for the peak region predicted by the mask generation network, indicating the probability that a specific sampling point belongs to a peak region.

#### 4.4.2. Evaluation Metrics

In this study, SNR, PSNR, and MSE were selected as evaluation metrics. The reference spectrum was obtained by averaging multiple high-SNR spectra of the same substance to eliminate random noise, followed by baseline correction.

MSE is used to evaluate the global deviation degree of Raman spectra after denoising, and its calculation formula is as follows:

(6) $$\mathrm{MSE}=\frac{1}{L}\sum_{i=1}^{L}{\left({\hat{y}}_{i}-y_{i}\right)}^{2},$$

where ${\hat{y}}_{i}\;$ is the denoised spectrum, *y_i_* is the reference spectrum, and *L* is the number of spectral sampling points. The smaller the MSE value, the more similar the structure of the denoised spectrum is to the reference spectrum.

SNR is used to evaluate the global noise level of Raman spectra, and its calculation formula is as follows:

(7) $$\mathrm{SNR}=10\log_{10}\left(\frac{\sum_{i=1}^{L}y_{i}^{2}}{\sum_{i=1}^{L}{\left({\hat{y}}_{i}-y_{i}\right)}^{2}}\right),$$

A higher SNR value indicates a greater ratio of effective signal to residual noise in the denoised spectrum, and a more significant denoising effect.

PSNR is used to evaluate the fidelity of Raman spectral peaks, and its calculation formula is as follows:

(8) $$\mathrm{PSNR}=10\log_{10}\left(\frac{\max{\left(y\right)}^{2}}{\mathrm{MSE}}\right),$$

A larger PSNR value indicates better fidelity to characteristic peaks.

At the same time, the classification accuracy of the KNN classifier is also used as an evaluation metric to verify the effectiveness of the DMPA-UNet denoising model on classification qualitative indicators.

## Figures and Tables

**Table 1:** Characteristic Raman peak positions of the six benzodiazepines.

Substance	Characteristic Raman Peaks (cm^−1^)
Diazepam (Dia)	1002, 1167, 1312, 1557, 1592
Flunitrazepam (Flu)	1100, 1170, 1337, 1578, 1611
Nimetazepam (Nim)	1002, 1067, 1340, 1578, 1614
Nordazepam (Nor)	867, 948, 1202, 1305, 1597
Oxazepam (Oxa)	854, 935, 1001, 1171, 1599
Temazepam (Tem)	689, 833, 1002, 1174, 1595

**Table 2:** Classification performance indexes under different SNRs.

SNR	Accuracy	Precision	Recall	F1-Score
High SNR	93.9%	94.2%	93.7%	93.8%
Low SNR	49.8%	51.8%	50.1%	49.1%

**Table 3:** Performance evaluation indexes of different noise reduction methods.

Methods	MSE	SNR (dB)	PSNR (dB)
Median Filtering	0.0110	7.7	20.2
SG Filtering	0.0102	8.1	20.6
CNN	0.0053	10.7	23.2
DMPA-UNet	0.0027	13.6	26.1

**Table 4:** Classification performance of different noise reduction methods.

Methods	Accuracy	Precision	Recall	F1-Score
Noisy	49.8%	51.8%	50.1%	49.1%
Median Filtering	65.4%	67.4%	65.1%	64.9%
SG Filtering	72.2%	72.9%	72.0%	72.1%
CNN	68.3%	68.4%	68.2%	67.8%
DMPA-UNet	84.5%	85.4%	84.2%	84.4%

**Table 5:** The number of recorded Raman spectra for each substance at different concentrations.

Concentration	Oxa	Dia	Flu	Nim	Nor	Tem	Total
1 × 10^−2^ ppm	58	51	54	52	51	54	320
1 × 10^−3^ ppm	67	61	52	50	51	56	337
1 × 10^−4^ ppm	53	57	59	55	54	55	333
1 × 10^−5^ ppm	57	55	56	57	52	52	329
1 × 10^−6^ ppm	51	53	67	52	50	54	327
Total	286	277	288	266	258	271	1646

## Data Availability

Dataset available on request from the authors.

## Supplementary Materials

The following supporting information can be downloaded at https://www.mdpi.com/article/10.3390/molecules31214030/s1, Figure S1: (A1–F1) Chemical structures of (A1) oxazepam, (B1) diazepam, (C1) flunitrazepam, (D1) nimetazepam, (E1) nordazepam, and (F1) temazepam. (A2–F2) Spectral comparison: Comparison of Raman, SERS, and DFT-calculated spectra for (A2) oxazepam, (B2) diazepam, (C2) flunitrazepam, (D2) nimetazepam, (E2) nordazepam, and (F2) temazepam. (A3–F3) Concentration-dependent SERS spectra: SERS spectra at concentrations ranging from 10−2 to 10−7 ppm for (A3) oxazepam, (B3) diazepam, (C3) flunitrazepam, (D3) nimetazepam, (E3) nordazepam, and (F3) temazepam. (A4–F4) Reproducibility (RSD) of (A4) oxazepam, (B4) diazepam, (C4) flunitrazepam, (D4) nimetazepam, (E4) nordazepam, and (F4) temazepam; Table S1: Identification of characteristic Raman peaks of oxazepam; Table S2: Identification of characteristic Raman peaks of diazepam; Table S3: Identification of characteristic Raman peaks of flunitrazepam; Table S4: Identification of characteristic Raman peaks of nimetazepam; Table S5: Identification of characteristic Raman peaks of nordazepam; Table S6: Identification of characteristic Raman peaks of temazepam.

## Abbreviations

The following abbreviations are used in this manuscript:

DMPA-UNet	dual-Mask peak-Aware U-Net joint denoising and baseline correction
SG	Savitzky–Golay
CNN	Convolutional Neural Network
SNR	Signal-to-noise ratio
PSNR	Peak signal-to-noise ratio
KNN	K-Nearest Neighbor
