# Supplementary material for: Research on Raman Spectral Denoising and Baseline Correction Method Based on Dynamic Mask Partition U-Net
Source: Molecules. 2026 Nov 9;31(21):4030. doi: 10.3390/molecules31214030 (PMC13764900; doi:10.3390/molecules31214030)
Supplement: Supplementary file 1 [file molecules-31-04030-s001.zip › molecules-4653786-supplementary.pdf]

Supplementary Materials

Research on Raman Spectral Denoising and Baseline Correction Method Based on Dynamic Mask Partition U-Net

Yihao Lin [1], Xiaoyue Xia [1], Yifan Zhang [1], Zheer Huang [2] , Xuqing Wang [2], Yingsheng He [3], Jiye Wang [1,*] and Yazhou Qin [1,*]

1 Key Laboratory of Drug Prevention and Control Technology of Zhejiang Province, Zhejiang Police College, 555 Binwen Road, Binjiang District, Hangzhou 310053, Zhejiang Province, China;

2 School of Pharmacy, Hangzhou Normal University, Hangzhou 311121, Zhejiang, China.

3 Key Laboratory of Drug Prevention and Control Technology of Zhejiang Province, National Narcotics Laboratory Zhejiang Regional Center, 555 Binwen Road, Binjiang District, Hangzhou, 310053, Zhejiang Province, China.

* Correspondence: yazhouqin@zju.edu.cn (Y. Z. Qin), wangjiye@zjjcxy.cn(J. Y. Wang)

Table S1. Identification of characteristic Raman peaks of oxazepam.

**Oxazepam**	**Raman Shift/cm^{-1}**	**DFT（Discrete Fourier Transform）/cm^{-1}**
ϱ(C-H)$_{benzene}$	854	848
ν(C-C),β(ring)	935	920
ν (C-CL),ν(C-N),ϱ(C-H)$_{benzene}$	1001	1020
ν(C-C)$_{benzene}$,ν(C-CL),ϱ(C-O),ν(C-C),ν(C-N),ϱ(C-H)	1171	1197
ν (C-C)$_{benzene}$,ν (C＝N),ϱ(C-H)$_{benzene}$	1599	1616

Table S2. Identification of characteristic Raman peaks of diazepam.

**Diazepam**	**Raman Shift/cm^{-1}**	**DFT/cm^{-1}**
ϱ(C-H)$_{benzene}$	1002	633
ϱ(C-H),ϱ(C-H)$_{benzene}$	1167	1165
ν (C-C),ν(C-N),ϱ(C-H)$_{benzene}$	1312	1310
ν (C-C)$_{benzene}$,ν(C-C),ϱ(C-H)$_{benzene}$	1557	1561
ν (C-C)$_{benzene}$,ν(C＝N),ν(C-N),ϱ(C-H)$_{benzene}$	1592	1603

Table S3. Identification of characteristic Raman peaks of flunitrazepam.

**Flunitrazepam**	**Raman Shift/cm^{-1}**	**DFT/cm^{-1}**
ν (C-N),ϱ(C-C)$_{benzene}$	1100	1097
ν(C-N),ν (C-C)benzene,ϱ(C-H)$_{benzene}$	1170	1184
ν (C-N),ν (C-C),ϱ(C-H)$_{benzene}$	1337	1339
ϱ(C-H)	1578	1544
ν (C-C)$_{benzene}$,ν(N=O),ν(C＝N),ϱ(C-H)$_{benzene}$	1611	1606

Table S4. Identification of characteristic Raman peaks of nimetazepam.

Nimetazepam	Raman Shift/cm^{-1}	DFT/cm^{-1}
ϱ(C-H)$_{benzene}$	1002	1004
ν (C-C)$_{benzene}$,ϱ(C-H)$_{benzene}$	1067	1061
ν(C-N),ϱ(C-H),ϱ(C-H)$_{benzene}$	1340	1344
ν(C-C),ν(C=N),ν(C-C)$_{benzene}$,ν(N=O),ϱ(C-H)$_{benzene}$	1578	1604
ν (N=O),ν (C-C)$_{benzene}$,ν(C=N),ϱ(C-H)$_{benzene}$	1614	1633

Table S5. Identification of characteristic Raman peaks of nordazepam.

Nordazepam	Raman Shift/cm^{-1}	DFT/cm^{-1}
ϱ(C-H)$_{benzene}$	867	864
ϱ(C-H)$_{benzene}$	948	946
ϱ(C-H)$_{benzene}$	1202	1203
ν (C-C),ν(C-N),ϱ(C-H),ϱ(C-H)$_{benzene}$	1305	1302
ν (C-C),ν (C-C)$_{benzene}$,ϱ(N-H),ϱ(C-H)$_{benzene}$	1597	1592

Table S6. Identification of characteristic Raman peaks of temazepam.

Temazepam	Raman Shift/cm-1	DFT/cm-1
ν(C-CL),ϱ(C-C)$_{benzene}$	689	693
ϱ(C-H)$_{benzene}$,ϱ(C-C)$_{benzene}$	833	840
ϱ(C-H)$_{benzene}$	1002	1004
ν (C-O),ϱ(O-H)	1174	1170
ν (C-N),ν (C-C)$_{benzene}$,ν(C=N), ϱ(C-C)$_{benzene}$	1595	1609

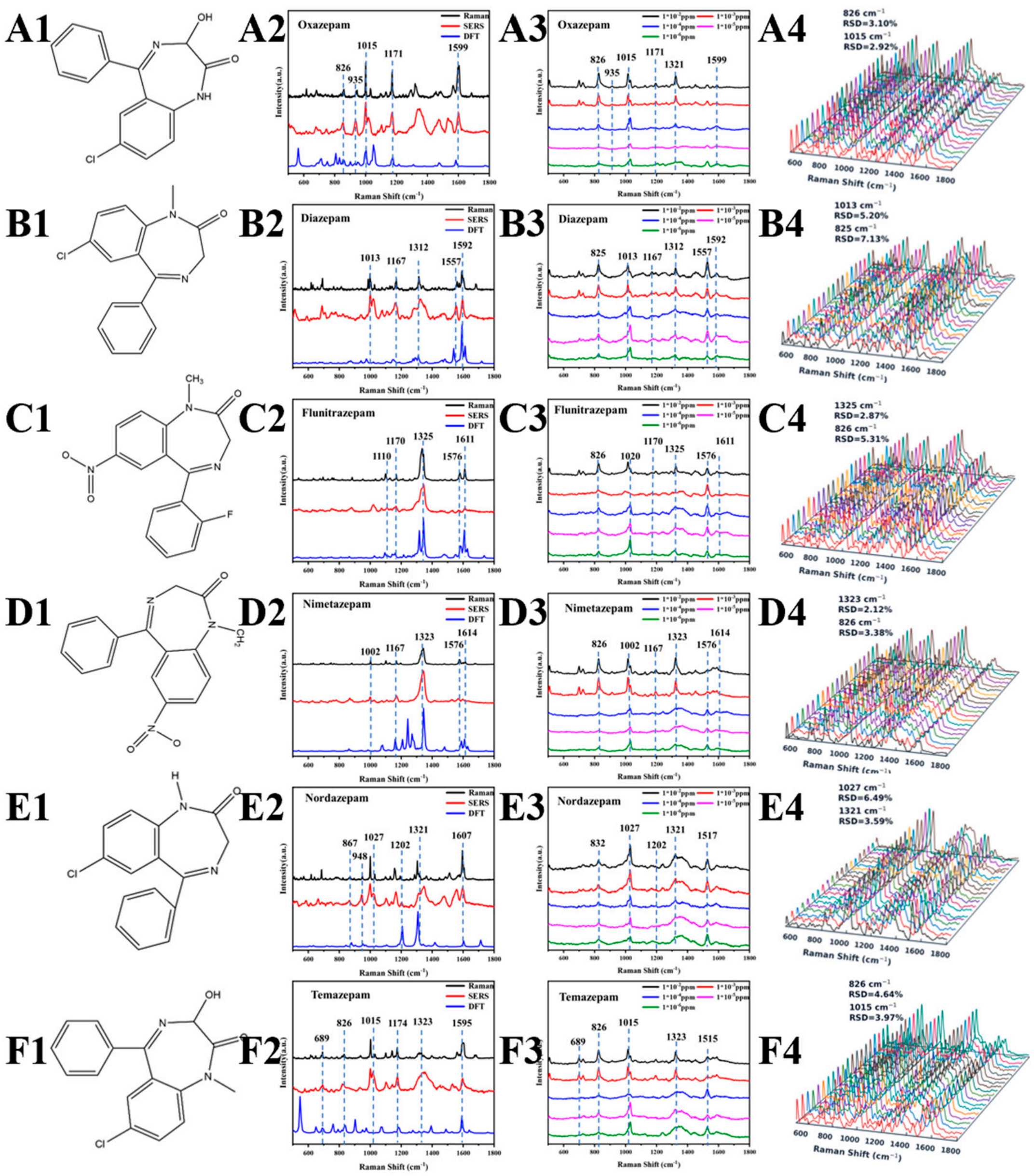

Figure S1. (A1–F1) Chemical structures of (A1) oxazepam, (B1) diazepam, (C1) flunitrazepam, (D1) nimetazepam, (E1) nordazepam, and (F1) temazepam. (A2–F2) Spectral comparison: Comparison of Raman, SERS, and DFT-calculated spectra for (A2) oxazepam, (B2) diazepam, (C2) flunitrazepam, (D2) nimetazepam, (E2) nordazepam, and (F2) temazepam. (A3–F3) Concentration-dependent SERS spectra: SERS spectra at concentrations ranging from 10^{-2} to 10^{-7} ppm for (A3) oxazepam, (B3) diazepam, (C3) flunitrazepam, (D3) nimetazepam, (E3) nordazepam, and (F3) temazepam. (A4–F4) Reproducibility (RSD) of (A4) oxazepam, (B4) diazepam, (C4) flunitrazepam, (D4) nimetazepam, (E4) nordazepam, and (F4) temazepam.
